# Diffusion Tensor Imaging as a Prognostic Tool for Recovery in Acute and Hyperacute Stroke

**DOI:** 10.3390/neurolint14040069

**Published:** 2022-10-21

**Authors:** Foteini Christidi, Dimitrios Tsiptsios, Aggeliki Fotiadou, Sofia Kitmeridou, Stella Karatzetzou, Konstantinos Tsamakis, Anastasia Sousanidou, Evlampia A. Psatha, Efstratios Karavasilis, Ioannis Seimenis, Christos Kokkotis, Nikolaos Aggelousis, Konstantinos Vadikolias

**Affiliations:** 1 Neurology Department, School of Medicine, Democritus University of Thrace, 68100 Alexandroupolis, Greece; 2 Institute of Psychiatry, Psychology and Neuroscience (IoPPN), King’s College London, London SE5 8AB, UK; 3 Department of Radiology, School of Medicine, Democritus University of Thrace, 68100 Alexandroupolis, Greece; 4 School of Medicine, Democritus University of Thrace, 68100 Alexandroupolis, Greece; 5 Medical Physics Laboratory, School of Medicine, National and Kapodistrian University, 11527 Athens, Greece; 6 Department of Physical Education and Sport Science, Democritus University of Thrace, 69100 Komotini, Greece

**Keywords:** diffusion tensor imaging, tractography, acute stroke, hyperacute stroke, stroke prognosis, biomarkers

## Abstract

Stroke represents a major cause of mortality and long-term disability among adult populations, leaving a devastating socioeconomic impact globally. Clinical manifestation of stroke is characterized by great diversity, ranging from minor disability to considerable neurological impairment interfering with activities of daily living and even death. Prognostic ambiguity has stimulated the interest for implementing stroke recovery biomarkers, including those provided by structural neuroimaging techniques, i.e., diffusion tensor imaging (DTI) and tractography for the study of white matter (WM) integrity. Considering the necessity of prompt and accurate prognosis in stroke survivors along with the potential capacity of DTI as a relevant imaging biomarker, the purpose of our study was to review the pertinent literature published within the last decade regarding DTI as a prognostic tool for recovery in acute and hyperacute stroke. We conducted a thorough literature search in two databases (MEDLINE and Science Direct) in order to trace all relevant studies published between 1 January 2012 and 16 March 2022 using predefined terms as key words. Only full-text human studies published in the English language were included. Forty-four studies were identified and are included in this review. We present main findings and by describing several methodological issues, we highlight shortcomings and gaps in the current literature so that research priorities for future research can be outlined. Our review suggests that DTI can track longitudinal changes and identify prognostic correlates in acute and hyperacute stroke patients.

## 1. Introduction

Stroke represents a major cause of mortality and long-term disability among the adult population, leaving a devastating socioeconomic impact globally. More specifically, in Western countries, up to 4% of healthcare resources are expended on stroke. In the United States, the mean stroke-related cost per individual, including rehabilitation, is nearly USD 140,000 [1]. Taking into account that stroke incidence is highly age-dependent along with the continuously increasing life expectancy, a substantial increase in the numbers of stroke survivors is anticipated [2,3]. In this context, efficient prognostication is of paramount importance, as prompt and precise identification of recovery capabilities may optimize treatment strategies in poststroke patients.

Clinical manifestation of stroke is characterized by great diversity, ranging from minor disability to considerable neurological impairment interfering with activities of daily living (ADL) and even causing death. Prognostic ambiguity has stimulated an interest in implementing stroke recovery biomarkers. Ideally, prognostic markers hold high sensitivity and specificity, enabling appropriate management of healthcare resources and individualization of rehabilitation treatments. Efficiency of the selected biomarker is also based on its capacity to accurately depict underlying mechanisms of disease. Moreover, it should be non-invasive, readily accessible to patients and clinicians, easily interpreted by physicians, reproducible, and cost-effective [4,5].

**Figure 1:**
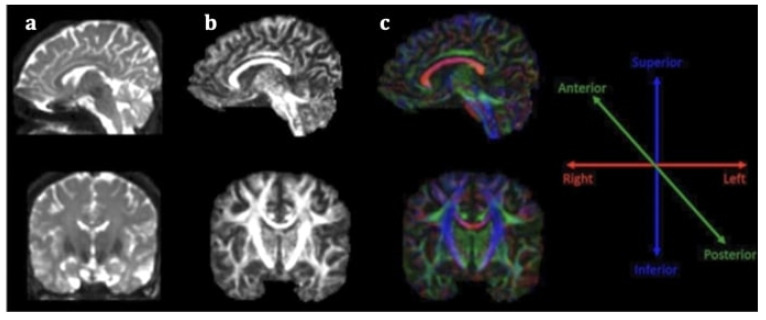
Sagittal (first line) and coronal (second line) section of DTI data (a), fractional anisotropy map (**b**) and color fractional anisotropy map (**c**). The color-coding of the white matter tracts in the color fractional anisotropy map follows the assumption: red for left–right-oriented fibers, blue for superior–inferior-oriented fibers and green for anteroposterior-oriented fibers.

Stroke diagnosis has been facilitated by the incorporation of advanced neuroimaging modalities in clinical practice, especially diffusion weighted imaging (DWI), a technique based on the motion of water molecules. Nevertheless, conventional magnetic resonance imaging (MRI) is incapable of accurately illustrating microstructural impairment on white matter (WM) tracts, rendering its role in predicting stroke outcome limited. Conversely, diffusion tensor imaging (DTI) is an extension of this modality for in vivo mapping of white matter (WM) directionality and organization, allowing the qualitative and quantitative evaluation of major WM tracts and their microstructural integrity [6]. DTI is based on the random diffusion of water molecules [7]. In WM, water diffusion is slower perpendicular to the fibers, but it occurs faster along their longitudinal axis, producing anisotropic diffusion. The extent of anisotropy is influenced by integrity and organization of the WM tract and water diffusion mobility generated by axonal membranes and their myelin sheaths. Different computational algorithms are used to track different WM bundles and study WM organization in healthy participants and different disease samples [8]. The most widely employed DTI parameters are fractional anisotropy (FA), mean diffusivity (MD), axial diffusivity (AD), and radial diffusivity (RD). FA measures the preferential directionality of diffusion and is quantitatively expressed in numerical values between 0 and 1. High FA values indicate a greater degree of preferential directionality (anisotropic diffusion). Such a high degree of preferential directionality is commonly observed in highly organized WM tracts. On the other hand, low FA values indicate less preferential directionality of water molecules (isotropic diffusion). Low FA values close to 0 are observed in gray matter and cerebrospinal fluid. MD indicates the diffusion magnitude, AD describes the diffusivity along the dominant diffusion direction, and RD portrays the average diffusivity of two shorter eigenvectors [7]. It has been suggested that AD is mostly related to axonal degeneration whereas RD is mostly linked to demyelinating processes [9]. DTI offers directional information on water molecule diffusion and provides additional maps, including the fractional anisotropy (FA) map and the color-coded directional map (Figure 1). The color-coded directional maps are based on the convention that the blue color represents the water molecules that diffuse in an inferior–superior direction, the green color represents that water molecules that diffuse in an anteroposterior direction, and the red color represents the water molecules that diffuse in a left–right direction. Although DTI has not yet been incorporated in routine clinical care in stroke, a growing body of research suggests that it is a promising imaging biomarker for stroke recovery, owing to its ability to imprint white matter tract integrity in detail [9].

**Figure 2:**
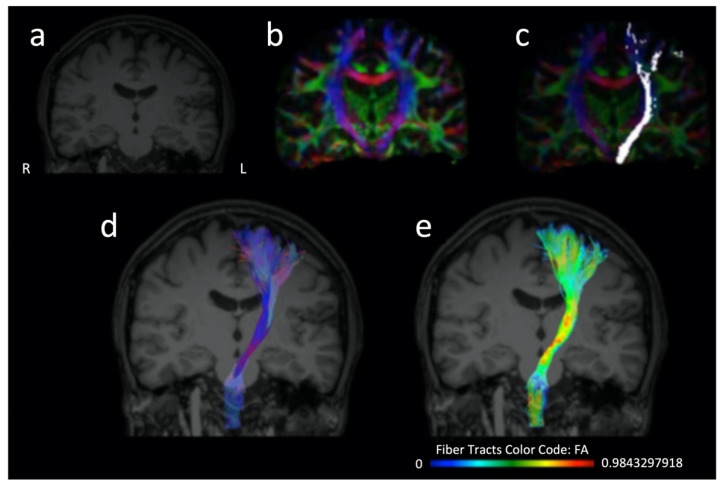
Three-dimensional T1 sequence (**a**), color fractional anisotropy map (**b**), distribution of the corticospinal tract fibers projected over a color fractional anisotropy map (**c**), three dimensional representation of the corticospinal tract (**d**,**e**) which is further color-coded according to the distribution of the fractional anisotropy values along the tract (**e**) and projected over a T1 sequence (**d**,**e**). The reconstruction of the corticospinal tract has been performed on a healthy subject (who provided written informed consent for the data acquisition, analysis and presentation) using the Brainance MD platform (Advantis Medical Imaging).

The majority of studies assessing DTI value as a stroke recovery biomarker have focused primarily on corticospinal tract (CST) integrity [6]. CST is a descending pathway (Figure 2) of great significance for motor function. Thus, it is reasonable that research on post-stroke rehabilitation has vastly focused on its assessment [10,11]. Among DTI parameters, FA is the most frequently utilized in research related to stroke recovery. Decrease of FA values of CST within the subacute stroke phase is widely associated with poorer motor outcomes [6,12,13]. Nevertheless, both increased and decreased FA values can be seen acutely [14]. Interestingly, it has been suggested that FA increase in the acute phase can be probably attributed to the more profound decrement in isotropic compared to anisotropic diffusion; therefore it cannot predict lesion age at the hyperacute stage [14]. Methods of FA of CST evaluation include measuring FA remotely at the stroke location, or the number of fibers passing through the stroke with tractography and calculating the ratio between the ipsilesional and contralesional pyramidal tracts [12]. Besides CST, DTI research has also expanded to other WM tracts, depending on the type of neurological deficit explored [15], including for example the common language-related WM network not only in the left but also in the right hemisphere [16,17,18,19,20].

Research on DTI metrics as stroke outcome biomarker is not limited to the acute and subacute phases, as it is also implemented on chronic stroke patients [13,21]. Direct visualization of long tracts and their potential disruption provides insight into pathogenesis of functional deficits in stroke survivors as well as compensatory mechanisms on a microstructural level. Such knowledge may elucidate which group of patients is most likely to benefit from rehabilitation, and even help personalize treatment plans after the acute stroke phase according to each individual’s needs. Of note, it has been evidenced that rehabilitative processes induce microstructural alterations reflected on DTI and are compatible with neural integrity [13]. 

Considering the necessity of prompt and accurate prognosis in stroke survivors along with the potential capacity of DTI as a relevant imaging biomarker, the purpose of our study was to review the pertinent literature published within the last decade regarding DTI as a prognostic tool in acute and hyperacute stroke.

## 2. Materials and Methods

The Preferred Reporting Items for Systematic Reviews (PRISMA checklist) was used to guide this study. Our study’s methods were designed a priori.

### 2.1. Research Strategy

**Figure 3:**
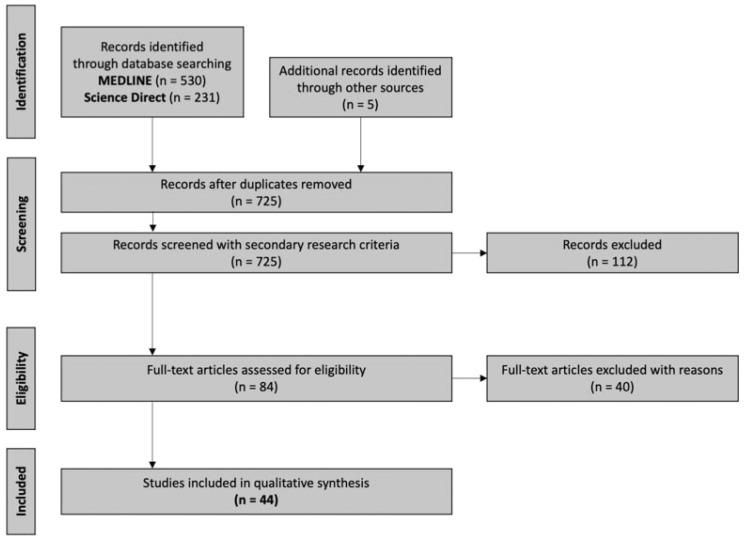
Study flow chart (PRISMA diagram).

A literature research of two databases (MEDLINE and Science Direct) was conducted by one investigator in order to trace all relevant studies published between 1 January 2012 and 16 March 2022, using either “diffusion tensor imaging” as a keyword or related term “DTI” as a search criterion. Moreover, the terms “stroke prognosis” or “stroke outcome” or “stroke recovery” were used as a second search criterion. The retrieved articles were also hand-searched for any further potential eligible articles. Any disagreement regarding screening or selection process was solved by a second investigator until consensus was reached. Figure 3 presents the review flowchart. 

### 2.2. Selection Criteria

Only full-text original articles published in the English language were included. Secondary analyses, reviews, case reports, guidelines, meeting summaries, comments, unpublished abstracts or studies conducted in animals were excluded. There was no restriction on study design or sample characteristics.

### 2.3. Data Extraction

Data extraction was performed using a predefined data form created in Excel. We recorded author, year of publication, number and age of participants, study design with regards to DTI acquisition (cross-sectional, longitudinal), type of stroke, time of DTI acquisition, main DTI parameters (MR field strength, diffusion directions, DTI analysis, and extracted parameters), anatomical regions examined, scales utilized, and main findings.

### 2.4. Data Analysis 

No statistical analysis or meta-analysis was performed due to the high heterogeneity among studies. Thus, the data were only descriptively analyzed.

## 3. Results

### 3.1. Database Searches

Overall, 767 records were retrieved from the database search. Duplicates and irrelevant studies were excluded; hence, a total of 84 articles were selected. After screening the full text of the articles, 44 studies were eligible for inclusion.

### 3.2. Study Characteristics

Forty-four publications fulfilled our inclusion criteria. They were classified into three groups, according to the type of stroke. The first group comprised 29 studies focusing on ischemic stroke (Table 1), the second group consisted of 11 studies focusing on hemorrhagic stroke (Table 2), while the third group embodied 4 studies, assessing the prognostic value of DTI in a group comprising both ischemic stroke and hemorrhagic stroke patients (Table 3). 

### 3.3. Study Design

Twenty-three studies were cross-sectional, and 21 studies were longitudinal with regards to the DTI acquisition. Of note, the majority of ischemic stroke studies were longitudinal (18/29 studies), the majority of hemorrhagic stroke studies were cross-sectional (8/11 studies) while all of the studies including both cohorts were cross-sectional (4/4 studies).

### 3.4. Stroke Patient Groups

The total number of stroke patients included in all studies ranged from *n* = 3 [22] to *n* = 165 [23]. Across the 44 studies, 6 studies had a disease sample size between 3–15 patients, 16 studies had a disease sample size between 16–30 patients, 11 had a disease sample size between 31–50 patients, and 11 studies had a disease sample size larger than 50 patients, with three of them including a disease sample size ≥100 patients [23,24]. 

### 3.5. Reference Groups

Across the 44 studies, the stroke patients were contrasted to demographically-matched healthy individuals in only 15 studies, with the rest of them (29/44 studies) not including a healthy control group (29/44 studies). None of the studies included a disease-control group other than stroke patients.

### 3.6. Demographic and Clinical Profiles

One study does did directly report participants’ age [23]. Mean/median patients’ age ranged from 47.5 years [25] to 72.7 years [25]. A comprehensive clinical description of participants’ demographic and clinical profiles is important for identifying meaningful imaging markers for stroke prognosis. The focus on clinical variables varied among the identified studies. 

### 3.7. Target Brain Region

Except for three whole-brain studies that enabled the evaluation of WM changes in both motor and non-motor WM tracts [26,27,28], most studies focused on motor-related pathways, i.e., the pyramidal tract. Non-motor WM tracts were examined in six studies [18,26,29,30,31]. Interestingly, only two of them were dedicated non-motor WM studies that examined language-related pathways [29,30]. 

### 3.8. Supporting Imaging and Neurophysiological Modalities

Three studies included additional imaging modalities, i.e., resting-state functional MRI (rs-fMRI) [26,32,33]. With regards to additional neurophysiological measures, only one study examined the functional integrity of the pyramidal tract using transcranial magnetic stimulation (TMS) [34]. 

### 3.9. Field Strength, Acquisition Parameters (directions), Post-Processing Techniques and DTI Parameters

Seventeen studies used 1.5 T (10 studies were published between 2012–2017 and seven studies were published between 2018–2022) and 26 studies used 3 T (17 studies were published between 2012–2017 and 9 studies are published between 2018–2022). One study does did provide information regarding field strength [30]. With regards to the number of directions, three studies used 6 directions (two in 1.5 T [34,35] and one in 3.0 T [36]), eight studies used 12 directions (four in 1.5 T [37,38,39] and four in 3.0 T [40,41,42,43]), three studies used 15 directions (two in 1.5 T [44,45] and one in 3.0 T [23]), three studies used 16 directions (one in 1.5 T [46] and two in 3.0 T [24,47]), one study in 1.5 T used 20 directions [31], one study in 3.0 T used 25 directions [48], eight studies used 30 directions (two in 1.5 T [49,50] and six in 3.0 T [18,29,33,51,52,53]), three studies in 1.5 T used 32 directions [54,55,56], one study in 3.0 T used 55 directions [57], and seven studies in 3.0 T used 64 directions [25,26,27,32,58,59,60]). Six studies did not provide information regarding diffusion directions. Among the studies that applied tractography, four studies reported the use of probabilistic tractography/atlases [23,50,51,61]. With regards to the analysis software, 12 studies used manufacturer-provided software whereas almost half of them (22/44 studies) used non-commercial software packages for academic institutions (e.g., FSL, ExploreDTI, DTI Studio). With regards to the reporting metrics, the majority of studies focused on FA and FA ratio (rFA) (affected/unaffected tract or region of interest [ROI]), whilst additional information regarding diffusivity values (i.e., MD, AD, and RD and/or MD, AD, and RD ratio) were only provided in five studies [30,52,58,61,62]. Of note, fiber number (FN) and fiber number ration (rFN) was also reported in some studies. Three studies used qualitative characterization of reconstructed tracts (i.e., disrupted, displaced, preserved or non-disrupted, partially disrupted, and fully-disrupted) according to which they further categorize their stroke cohorts [37,38,39]. 

**Table 1:** Basic characteristics and main findings of studies including patients with ischemic stroke.

1st Author (Year)	Type of Stroke, Study Design, Participants, Age (years)	Time of DTI Acquisition, Field Strength, DTI Parameters, DTI Analysis/Metrics, Additional Imaging/Electrophysiology	Anatomical Region Examined	Outcome Scale Utilized	Main Findings
Ali (2012) [46]	Ischemic Cross-sectional; 21 patients; Age 54.8 ± 8.61.	0.9–3 days; 1.5 T, 16 directions; ROI analysis and tractography, FA values, rFA.	CST	NIHSS within 1 month.	Decreased FA in affected areas compared to unaffected areas. In patients with rFA under 0.8 at admission, motor function showed poor recovery at day of discharge. The reduction in the FA values of the affected side was correlated with the degree of pyramidal tract involvements that were significantly correlated with the motor outcome on patient’s discharge day.
Kwon (2012) [56]	Corona radiata infarct; Cross-sectional; 71 patients; Age 56.01 ± 10.50.	The early scanning group (ES group) within 14 days and the late scanning group (LS group) 15–28 days; 1.5 T, 32 directions; ROI-based manual reconstruction of CST, grouping according to CST integrity.	CST	MI at onset and at 6 months.	Predictability of DTT for motor outcome was better in patients who were scanned later (15–28 days after onset) than in patients who were scanned earlier (1–14 days after onset).
Puig (2013) [44]	MCA ischemic stroke; Longitudinal 70 patients; Age 72 ± 12.	≤12 h, 3 days, and 30 days, 1.5 T, 15 directions;	CST	NIHSS, MI at 2 years.	Mean FA in affected CST at the pons on day 30 decreased progressively in line with increasing motor deficit at 2-year follow-up. Mean FA values in unaffected CST increased in line with increasing motor deficit. rFA values on day 30 decreased in line with motor deficit at 2 years. rFA on day 30 was the only independent predictor of long-term motor outcome.
Forkel (2014) [29]	First-ever left MCA infarct; Cross-sectional; 16 patients; age 63.39 ± 18.44.	Overall mean 10 ± 6 days; 3 T, 30 directions; Volume of left and right longitudinal segment, anterior segment, and posterior segment of the arcuate fasciculus.	Perisylvian language networks (long-segment, anterior segment, and posterior segment of the AF).	WAB 14 days, 6 months.	In the left hemisphere the only independent predictor of longitudinal aphasia was the lesion size. For the right hemisphere, age and volume of the long segment of the AF were predictors of longitudinal aphasia severity. Age, gender, and lesion size had an overall predictive power of 28% for longitudinal aphasia severity. Age, gender, lesion size, and volume of the right long segment of the AF had an overall predictive power of 57% for longitudinal aphasia recovery.
Groisser (2014) [62]	Ischemic MCA territory; Longitudinal; 10 patients; Age 52.6 ± 13.48; 12 healthy adults.	3 to 7 days, 1 to 2 mo, and 6 to 7 mo; 3 T; Difference between the ipsilesional CST relative to the contralesional CST in FA (ΔFA), AD (ΔAD), and RD (ΔRD) (positive values: stroke-induced increases of the ipsilesional relative to contralesional; negative values: stroke-induced decreases).	CST	Upper-limb section of the MI, NHPT 3 to 7 days (S1 acute), 1 to 2 months (S2, subacute), and 6 to 7 months (S3, chronic).	Significant loss in CST ΔFA and ΔAD at S1. Patients showed significant decreases in ΔFA and increases in ΔAD και ΔRD over time. S1 potential predictors (CST diffusion, respective motor function, and DWI lesion volume) and S2 motor outcomes: Acute loss in CST AD is a strong predictor of subacute (S2) grip and overall strength of the paretic upper limb. S1 potential predictors (CST diffusion, respective motor function, and DWI lesion volume) and S3 motor outcomes: the prognostic value of acute loss in CST AD (ΔAD) extends from the subacute to the chronic post-stroke period, and from gross to fine motor functions. S2 potential predictors (CST diffusion, respective motor function, and DWI lesion volume) and S3 motor outcomes: while subacute loss in CST FA does not improve prognosis of chronic grip and MI, it is a better prognostic indicator of chronic dexterity than the behavioral measure of dexterity.
Maraka (2014) [57]	Ischemic; Longitudinal; 23 patients; Mean age 66.7 ± 12.	3–7 days, 30 days, 90 days; 3 T, 55 directions; Multi-ROI approach with ROIs in corona radiata; PLIC, and CP bilaterally; number of virtual fibers of the ipsilesional (FNi) and contralesional (FNc) CST, fiber number ratio (FNr, FNi/FNc).	CST	UE-FM, motor items of the [mNIHSS] 3–7 days, 30 days, and 90 days.	Positive correlations between the FNr (FNi/FNc CST) and the UE-FM score at each phase of ischemic stroke. Negative correlations between FNr and mNIHSS at each phase of ischemic stroke. In both cases, the correlations became stronger from acute to subacute and chronic phases. The combination of acute NIHSS and FNr significantly predicted chronic UE-FM score.
Rong (2014) [22]	Medulla infarct; Longitudinal; 3 patients; Age 55, 54, 74.	Within days 7, 14, and 30; 3 T; FA and rFA (ipsilateral/contralateral to the infarct) for three ROIs placed along the pyramidal tract pathway; tractography of the CST using two ROIs	Medulla, CP, internal capsule, and CST.	FM, BI at each visit.	Patients 1 and 2 showed good motor recovery after 14 days, and the FA values of their affected CST were slightly decreased. The affected CST passed along periinfarct areas and tract integrity was preserved in the medulla. Patient 3 had the most obvious decrease in FA values along the affected CST, with motor deficits of the right upper extremity after 30 days. The affected CST passed through the infarct and was disrupted in the medulla.
Takenobu (2014) [28]	Ischemic; Longitudinal; 10 patients; Age 72.7 ± 6.4.	Within 2 weeks; and at 1 and 3 months; 3 T; TBSS and ROI analysis, FA.	TBSS with ROI analysis for significant clusters found in the TBSS.	FM within 2 weeks, and at 1 and 3 months.	Significantly increased FA in the red nucleus and dorsal pons in the ipsilesional side at 3 months. Significantly decreased FA in the ipsilesional internal capsule at all time points, and in the CP, corona radiata, and CC at 3 months. FA values of clusters in the red nucleus, dorsal pons, midbody of CC, and CB were positively correlated with recovery of motor function.
Feng (2015) [51]	Ischemic; Cross-sectional; 76 patients; Age 56.5 ± 14.8; 12 controls.	Acute phase; 3 T, 30 directions; Lesion load of the CST, probabilistic tractography on healthy controls, weighted CST-lesion load (wCST-LL).	CST	UE-FM Scale 2–7 days after stroke and 3 months.	Correlation between wCST-LL and motor impairment at 3 months measured by UE-FM scale. In the severely impaired subgroup (defined as UE-FM ≤10 at baseline), wCST-LL correlated with outcomes significantly better than clinical assessment.
Liu (2015) [25]	Ischemic subcortical (first infarction); Longitudinal; 18 patients; Age median 47.5; 18 controls; Age median 49 years.	1, 4, 12 weeks; 3 T, 64 directions; FA maps, longitudinal changes in CST after stroke, voxel-based correlation analysis between FA changes and FM scores over time.	CST	FM, NIHSS	Four weeks after stroke onset, FA values in the CST located in the ipsilesional PMA and pons of patients were significantly decreased compared to controls and to FA values obtained 1-week post-stroke. Twelve weeks after stroke onset, significantly decreased FA values in the WM of the ipsilesional SMA of patients compared to the values 4 weeks post-onset. No significant FA changes in the contralesional CST between patients and controls during the entire follow-up period. Significantly positive correlations between FA values and FM scores within 12 weeks after subcortical infarction were found contralesionally in the medial frontal gyrus and thalamocortical connections. Twelve weeks post-stroke, FA values for the contralesional MFG and contralesional thalamocortical connections were significantly increased compared to FA values of controls and those of one week post-stroke. FM scores were positively correlated with FA values in the MFG and thalamocortical connections across the three time points after stroke.
Moulton (2015) [52]	Thrombolysed ischemic stroke of carotid artery territory; Cross-sectional; 28 patients; Age mean 68.5 ± 16.	24 h; 3 T, 30 directions; rFA, rMD, rAD, and rRD.	Subcortical WM of PrCG, corona radiata, PLIC, CP in ipsilesional and contralesional hemisphere; CCg as control region.	NIHSS day 1, 7, and mRS at 3 months.	Decreased MD and AD in corona radiata, PLIC, and M1 and decreased FA in corona radiata and M1 (affected vs. non-affected). Lower corona radiata rAD and corona radiata rMD were associated with a poorer outcome. The corona radiata rAD was the strongest independent predictor of the clinical scores (NIHSS on day 7 and mRS at 3 months) after adjusting for total infarct volume. Significant differences in corona radiata rAD between patients with complete recanalization and those without.
Zhang (2015) [60]	Pontine infarct; Longitudinal; 17 patients; Age mean 58.3; 17 controls.	Within 7, 14, 30, 90, and 180 days; 3 T, 64 directions; ROI analysis and tractography, FA and rFA (ipsilateral/contralateral).	ROIs: medulla, CP, internal capsule, CSO; tractography: CST.	FM, mRS, and BI.	rFA in the CST above the pons decreased significantly compared with those in the contralateral side and those in control subjects within 7 days, on day 14, and on day 30 after onset. rFA above the pons on day 14 correlated positively with the FM scores on day 90 and day 180 and correlated negatively with the mRSscore on day 90 and day 180. Follow-up tractography showed regeneration and reorganization of the motor pathways.
Bigourdan (2016) [24]	Minor-to-severe supratentorial cerebral infarct; Longitudinal; 117 patients; Age: 67 (58–78).	24–72 h, 1 year; 3 T, 16 directions; Deterministic tractography, semiautomatic calculation of stroke volumes, total N of fibers ipsilateral to stroke normalized by the total N of fibers from the contralateral side, iFNr and fFNr (fiber N ratio).	CST	FMA score at 1 year.	The iFNr measured at 24 to 72 h was strongly correlated to the fFNr measured at 1 year. iFNr correlated strongly with motor recovery (improving prediction compared with using only initial FMA, age, and stroke volume).
Doughty (2016) [49]	First-time ischemic hemispheric stroke; Cross-sectional; 58 patients; Age 61.3 ± 14.2; 12 controls.	Within 80 h from onset; 1.5 T, 30 directions; Lesion maps, FA, ADC and FLAIR values, FA LI (stroke affected vs. stroke unaffected hemisphere).	CP, a stretch of the CST caudal to each stroke lesion (Nearest-5-Slice, N5S).	UE-FM assessment in the acute phase and at 3 months.	The slope of the FA LI for the N5S ROI (S-N5S) showed a weak, significant trend as a predictor of 3-mo UE-FM score. The slope of the ADC LI for the N5S ROI was also weakly predictive of 3-month outcome. Initial UE-FM and wCST lesion load were strong predictors of 3-mo outcome.
Jang (2016) [55]	Pontine infarct; Cross-sectional; 31 patients; Age 64.76 ± 10.76.	7–28 days; 1.5 T, 32 directions; Multi-ROI tractography, FA, fiber number (FN), CST area size, rFA, rFN and rCST area (affected/unaffected).	CST	MI, MBC, and FAC within 24 h and at 6 months.	Significant correlation between rFN and rCST area and all 6-month motor outcome. No significant correlation between the rFA and all 6-month motor functions.
Liu (2017) [27]	Ischemic, subcortical; Longitudinal; 50 patients; Age 24–72, median 53.5; 2 controls; Age 32–71, median 52.7.	1, 4, and 12 weeks; 3 T, 64 directions; TBSS.	Whole-brain WM analysis using TBSS.	FM	No significant changes in FA, MD and LDH in WM tracts at week 1 between the PROP group and controls. No significant differences in FA in the POOR group relative to control and PROP groups. Subjects in the POOR group had reduced MD and LDH compared to controls in ipsilesional superior corona radiata (SCR), PLIC, external capsule, posterior corona radiata, ALIC, anterior corona radiata, CP, retrolenticular part of the IC. Subjects in the POOR group showed lower MD in an ROI encompassing ipsilesional SCR, ALIC, posterior corona radiata, anterior corona radiata, and SLF compared to PROP group 1 week after stroke. Subjects in POOR group had lower LDH values in a ROI encompassing ipsilesional PLIC, SCR, and retrolenticular part of the IC compared to PROP group 1 week after stroke. Initial FMA and LDH in the ipsilesional CST in the superior corona radiata and PLIC were predictors of motor improvement within 12 weeks after stroke.
Liu (2018) [58]	Ischemic, subcortical; Longitudinal; 22 patients; Age 40–75, median 51.6; 22 controls; Age 40–71, median 50.7.	1, 4, and 12 weeks; 3 T, 64 directions; FA, MD, AD, and RD for 4 ROIs (bilateral primary motor area [PMA] and cerebral peduncle [CP]), delta diffusion metrics (d12-d initial).	Bilateral PMA and bilateral CP.	FM	AD in the ipsilesional PMA and CP was lower at W1 in both PROP and POOR groups compared to controls and values in the contralesional PMA and CP. AD in the ipsilesional PMA in both PROP and POOR groups significantly increased at W4 and W12 compared to AD at W1, but no significant changes in AD in the ipsilesional CP at W4 and W12 compared to W1. Significant decreases in FA and increases in RD and MD in WM of the ipsilesional PMA and CP in both PROP and POOR groups at W4 and/or W12 compared to W1 and those in contralesional areas. For all of the patients, ΔFMA-UE-observed was greater in patients with higher ΔAD in the ipsilesional PMA. For PROP patients, only FMA-UEii predicted ΔFMA-UE-observed. For POOR group, only lesion volume was related to ΔFMA-UE-observed.
Liu (2018) [59]	Thalamic infarct; Longitudinal; 12 patients; Age 50.33 ± 7.62; 12 controls; Age 51.47 ± 8.43.	1 week, 4 weeks, 3 months and 6 months; 3 T, 64 directions; FA and MD for each ROI (ipsilesional and contralesional side for patients, mean values for both sides for controls).	Regions in corona radiata pathway: thalamus, corona radiata, and CSO.	NIHSS, BI, and NIHSS8.	Gradual increase of FA values in ipsilesional and contralesional thalamic radiation fibers from W1 to W6. No changes in controls. Negative association between FA increases and NIHSS and NIHSS8 decrease. Positive association between FA increases and BI increases.
Etherton (2019) [61]	Ischemic Longitudinal; 42 patients; Age 70.2 ± 9.2, with (*n*= 22) and without (*n* = 20) early neurological improvement.	Within 12 h and 3–5 days post stroke; 1.5 T (acute phase), 3 T follow-up (not for DTI); WMH and chronic strokes, acute infarct volume on DWI, PWI-DWI mismatch ration, FA, MD, RD and AD in WMH and NAWM contralateral to the acute infarct (probabilistic ICBM-atlas).	WMH and NAWM contralateral to the acute infarct.	NIHSS Admission, day 3–5 post-stroke.	NAWM RD and MD values were lower in the early neurological improvement + group compared to the early neurological improvement − group. Increasing NAWM RD decreased the likelihood of early neurological improvement. NAWM MD was an independent predictor of early neurological improvement (multivariate logistic regression).
Kulesh (2019) [63]	Ischemic; Cross-sectional; 100 patients; Age 68.3 ± 11.1; 10 controls.	5–10 days; 1.5 T, 12 directions; ROI analysis and tractography, FA values, and rFA.	CST (level of PLIC, pons), GIC, ALIC, CB, SLF, IFOF, SCC, infarction and the area within 3 cm from it.	Measures on day 3, 10, and at discharge: NIHSS, Frenchay Arm Test, BBS, HAI, RMI, MoCA, FIM, and mRS.	The indices of FA of the ipsilateral upper SLF and CB, FA and the size of the infarct focus, rFA of the CB, CST and the ALIC, as well as the FA of the splenium and the knee of the intact hemisphere were the most valuable predicting factors of functional outcome of acute IS. The integrity of the associative tracts of the affected hemisphere was more valuable than the microstructure of the intact hemisphere and rFA for predicting global outcome. The tracts of the intact hemisphere were more important for the restoration of complex rehabilitation spheres, such as cognitive status and daily living and social skills, which ensure patients’ independence.
Mahmoud (2019) [37]	Ischemic; Cross-sectional; 60 patients; Age 24–75, mean 58.2.	Within 2 days; 1.5 T, 12 directions; FA of WM tracts.	3D fiber tractography with multi-ROI technique and regions drawn in the unaffected portion of the WM tracts at the side of infarction and corresponding area at the contralateral hemisphere; degree of FA reduction of WM tracts at the site of infarction [mild (0.4), moderate (0.2–0.3), severe (0.1)]; and classification of WM tracts as disrupted, displaced, and preserved.	NIHSS at admission and after 3 months.	Residual neurological deficits in patients with disrupted tracts. Near-complete clinical recovery was found in patients with non-disrupted tracts. Significant association between the degree of FA reduction in the affected tracts and the clinical score at admission and the clinical recovery after 3 months.
Moulton (2019) [18]	Thrombolysed ischemic stroke of carotid artery territory; Cross-sectional; 45 patients (17 motor, mean age 65.5, and 28 aphasia, mean age 69.6).	24 h; 3 T, 30 directions; Tractography with AD and rAD (affected/unaffected), asymmetry maps for voxel-based analysis.	Second and third branches of the SLF (SLF-II and -III, respectively) and CST as part of the motor network in addition to the left and right AF, IFOF, ILF, and UF.	NIHSS, JTT, and AHS at 3 months.	Significant differences in AD between affected and unaffected tracts with the exception of the ILF in the language cohort. rAD of the CST was the sole independent predictor in the DTI model explaining 70.1% of the variance in motor impairments at 3 mo. rAD in AF—along with age and initial aphasia severity—was an independent predictor of 3-month aphasia outcome. These tract-specific results were confirmed in voxel-based analysis without a priori assumptions.
Keser (2020) [30]	Left hemispheric ischemic stroke; Longitudinal; 24 patients; Age 36–86.	Within 2 weeks and 6–12 months; DTI (no other information are available); Reconstruction of FAT and AF, FA and RD, WM lesions, actual volumes.	AF and FAT	BNT within 2 weeks and 6–12 months.	Acute phase: no lateralization between left and right AF and FAT (FA, RD). Chronic phase: Diminished FA with time in both hemisphere which was more pronounced for left FAT compared to right. The recovery rate did not correlate with acute right and left DTI values of AF and FAT. Longitudinal FA of the right AF was a significant predictor of naming recovery.
Berndt (2021) [23]	Acute occlusions of a large intracranial vessel of the anterior circulation who underwent mechanical recanalization; Cross-sectional; 165 patients; No demographics.	Median, 3 days; interquartile range [IQR], 3–4 days; maximum, 7 days; 3 T, 15 directions; Probabilistic tractography, FA [FA index = (FA_I_ − FA_H_)/(FA_I +_ FA_H_); I = infarcted, H = healthy non-affected.	CST (PLIC, PED)	NIHSS, mTICI, mRs at 90 days.	FA index of PLIC showed weak negative correlations to the NIHSS at the time of MR imaging. FA index was reduced in the acute poststroke phase with a correlation to clinical presentation, especially in case of peripheral infarcts. For peripheral infarcts, a strong effect of the FA index on clinical outcome (mRS) existed.
Darwish (2021) [54]	Supratentorial ischemic stroke; Longitudinal; 30 patients; Age 61.32 ± 4.34.	Time of admission and 1 month; 1.5 T, 32 directions; FA, rFA, MD, and FN.	CST (pons)	NIHSS at admission, and after 1, 6, and 9 months.	A significant negative correlation was found in moderate and severe motor deficit between rFA and FN in the ipsilateral CST at the rostral part of pons after 1 month of infarction and NIHSS score at 6 months.
Liu (2021) [32]	Ischemic subcortical, supratentorial; Longitudinal; 33 patients; Age 53.4 ± 1.81; 33 controls; Age 53.7 ± 2.32.	1, 4, and 12 weeks; 3 T, 64 directions; ROI analysis with mean FA and MD values.	Bilateral inferior cerebellar peduncle (JHU-ICBM-DTI-81-WMPM-90p).	FM	Decreased FA and increased MD in contralesional ICP at W12 after stroke compared with controls and with values at W1. Decreased FA but not MD changes in ipsilesional ICP at W4 and W12 compared to controls. Changes in FM-LL scores of the affected limb correlated positively with FA changes and negatively with MD changes in the contralesional ICP.
Xia (2021) [33]	Ischemic stroke of internal capsule and neighboring regions; Longitudinal; 34 patients; Age mean 63.4 34 controls; Age mean 61.2.	Weeks 1, 4, and 12; 3 T, 30 directions; Tractography with multi-ROIs, FA values of CST from PLIC; rsfMRI data for FC.	CST	NIHSS, MMSE, FMA, and BI after each scan.	FA values in the ipsilesional remaining CST showed a decline during the first week and increased longitudinally over the next 12 weeks after stroke. No FA changes in contralesional CST from week 1 to week 12 after stroke. Positive correlation between percentage changes of FA in ipsilesional remaining CST and those of interhemispheric FC in patients during weeks 1 to 4 after stroke. The increase of both interhemispheric FC and ipsilesional CST-FA were significantly correlated with the greater change of FMA between weeks 1 and 4 after stroke. Only increased FA of ipsilesional CST was significantly correlated with the greater change of FMA at weeks 4–12 after stroke compared to interhemispheric FC.
Li (2022) [26]	Pontine infarction; Longitudinal; 16 patients; Age 56.9 ± 7.7; 16 controls; Age 54.3 ± 5.2.	1 week (T1), 1/2 months (T2), 1 month (T3), 3 months (T4), and 6 months (T5) after onset; 3 T, DTI 64 directions; ROI FA analyses; rs-fMRI.	ATR, CST, CCG, CH, FMAJ, FMIN, IFOF, ILF, SLF, UF and SLF-TP.	UE-FM before and after each scan.	The FA values (TBSS) were significantly lower in the pontine infraction group than in the control group at T1, tended to expand at follow-up until the completely disappeared at T5. The FA values of the ATR, CST, CCG, FMAJ, FMIN, IFOF, ILF, SLF, UF, and SLF-TP were significantly increased at T2 compared with that at T1. There were no significant differences in the FA values of any specific ROIs among T3, T4, and T5.
Shaheen (2022) [38]	MCA ischemic stroke Longitudinal; 34 patients; Age 64.4 ± 6.7; 17 controls; Age 61.8 ± 5.2.	Baseline (within 7 days?) and 6 months; 1.5 T, 12 directions; CST tractography with multi-ROIs (anterior mid-pons, CP, PLIC, and corona radiata), FA and rFA, tract categorization as intact, displaced, and disrupted.	CST	NIHSS, MRC, mRS, and MI at baseline and 6 months.	Reduced baseline FA values of the CSTs on the affected side compared to the contralateral and controls. Lower mean baseline FA lesion side and rFA compared to follow-up. Patients with high baseline FA and rFA showed good recovery response. FA on the lesion side and rFA were negatively associated with follow-up NIHSS and MRS scores and positively associated with follow-up MI scores.

**Table 2:** Basic characteristics and main findings of studies including patients with hemorrhagic stroke.

1st Author (Year)	Type of Stroke, Study Design, Participants, Age (years)	Time of DTI Acquisition, Field Strength, DTI Parameters, DTI Analysis/Metrics, Additional Imaging/Electrophysiology	Anatomical Region Examined	Outcome Scale Utilized	Main Findings
Kuzu (2012) [36]	Hemorrhagic; Longitudinal; 23 patients; Age 65.26 ± 12.70.	Within 3 days, day 14, 30, 60, and 90; 3 T, 6 directions; FA values of bilateral CP.	Bilateral CP	NIHSS at day 90.	The mean FA value on day 3 was significantly higher in the good recovery group than in the poor recovery group. The mean FA value gradually decreased until day 90 in the poor recovery group, but not in the good recovery group. The FA value and the motor function score on day 3 were independent factors for predicting the motor function outcome. FA value on day 3 could predict motor function outcome with a sensitivity of 100% and a specificity of 77.8% at an FA value of 0.7 on day 3.
Wang (2012) [64]	ICH of putamen, thalamus, with or without intraventricular bleeding; Cross-sectional; 27 patients; Age 60.4 ± 10.7.	Either within 3 days or at 2 weeks after onset; 1.5 T; Average FA and MD of 6 manual regions manually drawn unilaterally in the anterior CP, and rFA.	CP	mRS, FIM, NIHSS, and PG at 6 monhts.	The FA values within 3 days and after 2 weeks of ICH onset were significantly decreased at the affected side, but the mean MD remained unchanged. The rFA within 3 days was negatively correlated with the PG, positively correlated with the FIM scores, and negatively correlated with the mRS scores at the end of follow-up. The rFA at 2 weeks was positively correlated with the FIM and negatively correlated with mRS scores and PG at the end of follow-up. A cutoff point of 0.955 for rFA within 3 days had 53% sensitivity and 100% specificity for predicting good motor outcome over 6 months. A cutoff point of 0.875 for rFA values obtained after 2 weeks had a sensitivity of 76% and a specificity 89% for predicting good motor outcome over 6 mo.
Koyama (2013a) [43]	Thalamic and/or putaminal hemorrhage; Cross-sectional; 32 patients; Age 64.25 ± 13.43.	Days 14–18; 3 T, 12 directions; FA values (left and right ROIs), rFA.	CP and the corona radiata/internal capsule.	MRC at 1 month.	Cerebral peduncle rFA values had statistically significant relationships with MRC scores. rFA values for the corona radiata/internal capsule showed less significant relationships.
Koyama (2013b) [41]	Thalamic and putaminal hemorrhage; Cross-sectional; 12 patients; Age 62.92 ± 14.64.	14–18 days after admission; 3 T, 12 directions; FA values (left and right ROIs), rFA.	CP	BRS, FIM-motor at 3–7 months.	High statistically significant relationship between rFA and upper extremity function. Medium statistically significant relationship between rFA and lower extremity function. Analysis of rFA and FIM-motor scores did not reveal statistical significance.
Ma (2014) [35]	Hemorrhagic (basal ganglia); Longitudinal; 23 patients; Age 34–67, mean 54 ± 9.	Day 0, 30, and 90; 1.5 T, 6 directions; Seed ROI in the CST portion of the ipsilesional CP	CST	MFS on day 90.	Mean FA value on day 0 was significantly lower in the poor outcome group compared to the good outcome group. FA value gradually decreased in the poor outcome group until day 90 after onset while it continously increased in the good outcome group. The MFS obtained at day 90 after onset was significantly correlated with the initial FA value in the affected cerebral peduncle. FA value on day 0 could predict motor function outcome with a sensitivity of 88.89% and specificity of 92.86% at the initial FA value of 0.45.
Tao (2014) [50]	Hemorrhage; Cross-sectional; 32 patients with CST affected by hematoma; Age 65.59 ± 17.09; 12 controls.	4 days after ICH onset; 1.5 T, 30 directions; FA in the affected and unaffected side at nearest slices under the lesion and at CP, rFA in the CP, mean rFA of “consecutive 5 slices” under the hematoma, and rFA in 5 individual slices.	CST	mRS at follow-up visits in the outpatient clinic.	The rFA measured in the cerebral peduncle, but not the 5 slices below the lesion, was significantly lower in the group with poor functional outcome than those with good functional outcome. The ICH score had greater areas under ROC curve in predicting functional outcome compared to the mean rFA
Cheng (2015) [53]	Hemorrhage; Cross-sectional; 48 patients; Age 62 ± 14.	Within 14 days (mean 7 days); 3 T, 30 directions; FA, ADC, rFA, rADC, and CST tractography-based groups: preservation around the haematoma (type A), partial interruption (type B), and complete interruption at or around the haematoma (type C).	Pons, CP, perihaematoma oedema, and corona radiata.	MI at admission, at 1 and 3 months.	14 patients in type A, 20 patients in type B, 14 patients in type C. No significant differences on rFA between groups on pons, CP, oedema except for corona radiata. No significant differences on rADC between the three groups on four ROIs. The rFA at the corona radiata was significantly positively correlated with the MI at admission, at 1 month and 3 months. No correlation between rADC and motor functions at any time.
Koyama (2015) [42]	Putaminal and/or thalamic hemorrhage; Cross-sectional; 40 patients; Age 61.83 ± 12.85.	14–21 days after admission; 3 T, 12 directions; FA values of left and right ROIs, rFA.	CP	BRS (shoulder/elbow/ forearm, hand, lower extremity), FIM-motor, and length of total hospital stay from admission to acute medical service to discharge from long-term rehabilitation facility (LOS).	Moderate-to-high positive correlations between rFA and S/E/F BRS, hand BRS, LE BRS, and FIM-motor (though weaker). Negative moderate correlation between rFA and LOS. Logistic model fit was moderate for shoulder, elbow, forearm BRS and lower extremity BRS and much higher for hand BRS.
Fragata (2017) [31]	Acute spontaneous SAH; Cross-sectional; 60 patients; Age 59 (35–86).	<72 h; 1.5 T, 20 directions; FA and ADC averaged between left and right ROIs, whole brain FA and ADC.	Frontal CSO, parietal CSO, lentiform nucleus, thalamus, PLIC, CCg, CCs, and mid-pons cerebellar peduncles.	mRS, presence of DCI at 3 months.	FA values in the cerebellum showed an association with DCI (multivariate analysis). No association between early ADC values and occurrence of DCI (multivariate analysis). Weak evidence of an association between ADC values at the frontal centrum semiovale and mRS at 3 months.
Min (2020) [48]	Putaminal hemorrhage; Longitudinal; 12 patients; Age 50 ± 12.	Within 1 day, 3 weeks, 3 months, and 6 months; 3 T, 25 directions; CST.	CST with ROIs at the pons.	BMS, MBI, mRS, NIHSS, JHFT, and MI.	The mean FA value on pons in the affected side of the brain was significantly higher in the good outcome group compared to the poor outcome group at baseline and 6 months after initial treatment. No significant differences in mean FA value in the non-affected side between the good and the poor outcome groups at all time points. The rFA of the good outcome group was significantly higher compared to the poor outcome group at baseline and 6 months after initial treatment. FA values and rFA at baseline significantly correlated with the sum of BMS scores 6 months after initial treatment.
Gong (2021) [45]	Hemorrhage; Cross-sectional; 75 patients; Age 58.8 ± 13.9.	Around 3 weeks after stroke; 1.5 T, 15 directions; ROI-based reconstruction of CST and qualitative data for no disruption, partial disruption, complete disruption of CST.	CST connecting the hand–knob area of the PrCG and the CP.	BRS-H at post-stroke 3 weeks and 3 months.	Degrees of integrity of the CST was negatively correlated with the BRS-H at post-stroke both 3 weeks and 3 months. Patients with intact CST or completely disrupted CST shown by DTI did not show significant improvement in the BRS-H at post-stroke 3 months. Patients with partially disrupted CST showed significant improvement in the BRS-H at post-stroke 3 months compared to 3 weeks.

**Table 3:** Basic characteristics and main findings of studies including patients with both ischemic and hemorrhagic stroke.

1st Author (Year)	Type of Stroke, Study Design, Participants, Age (years)	Time of DTI Acquisition Field Strength, DTI Parameters, DTI Analysis/Metrics, Additional Imaging/Electrophysiology	Anatomical Region Examined	Outcome Scale Utilized	Main Findings
Imura (2015) [47]	Ischemic (*n* = 16) and hemorrhagic (*n* = 9); Cross-sectional; 25 patients; Age 71.5 ± 11.	Within 10 days; 3 T, 16 directions; FA and ADC, FN asymmetry index.	CST	MI, BRS, BI, and FIM on the same data as DTI and at 1 month.	Significant correlation between the FA value of the affected CST and motor outcome (BRS-UE, BRS-F, BRS-LE, MI-UE, and MI-LE) within 10 days and 1 mo post-onset. Significant correlation between the FA value of the affected CST and ADL function (BI, BI-gait, FIM-M, and FIM-gait) within 10 days and 1 month post-onset.
Nakashima (2017) [39]	Ischemic (*n* = 12) and hemorrhagic (*n* = 5); Cross-sectional; 17 patients; Age mean 68 (40–93).	14 days after admission; 1.5 T, 12 directions; FA and rFA for ROIs placed in CP, pattern of tractography [incompletely disrupted-type A, completely disrupted-type B]; Additional VBM on 3D T1 using SPM12.	CP	FMA, MAL at 3 months.	12 patients as type A and 5 patients as type B. CP rFA was significantly correlated with FMA, amount of use, and quality of movement 3 months after stroke for type A.
Koyama (2018) [40]	Ischemic (*n* = 40) and hemorrhagic (*n* = 40); Cross-sectional; 80 patients; Age 31–89 for hemorrhage, 41–84 for infarct.	14–21 days after admission; 3 T, 12 directions; FA in the unaffected hemisphere, and rFA.	CP	BRS, FIM-motor monthly, and LOS.	Lower rFA in hemorrhage compared to the infarct group. The correlations between rFA and outcome measures were all statistically significant for both the hemorrhage and the infarct groups. The correlation patterns for BRS and LOS were very similar between the hemorrhage and the infarct groups.
Okamoto (2021) [34]	Ischemic (*n* = 8) and hemorrhagic (*n* = 9); Cross-sectional; 17 patients; Age 32–75.	25–64 days post-onset; 1.5 T, 6 directions; FA and rFA; Additional MEP and CMCT.	PLIC	FMA, ARAT, and use or non-use of a short leg brace at discharge from the recovery rehabilitation unit.	Positive correlation between rFA and FMA-UE, as well as ARAT score (better recovery of upper limb function).

## 4. Discussion

A literature review over the last decade was carried out in order to delineate DTI prognostic value in post-stroke patients. Forty-four full-text original articles assessing the potential applicability of DTI on stroke prognosis were identified and classified into three groups based on the type of stroke evaluated (ischemic, hemorrhagic, or both).

### 4.1. Prediction of Recovery Using Different DTI Parameters in Studies with Ischemic Cohorts: The Role of FA

Regarding the role of FA, Berndt and colleagues [23] studied 165 large-vessel occlusion stroke patients who underwent mechanical thrombectomy and DTI within 7 days post-stroke. They reported reduced FA of posterior limbs of the internal capsule (PLIC) in the acute phase, reflecting impairment of CST integrity, and weak negative correlation with the National Institutes of Health Stroke Scale (NIHSS) at time of MRI. Furthermore, FA within PLIC in the acute phase was significantly correlated with clinical outcome at 90 days in peripheral infarcts, whereas no significant association was found for basal ganglia infarcts. Similarly, Xia and colleagues [33] reported a decline in FA values in the ipsilesional CST during the first week poststroke and then longitudinal increase over the next 12 weeks. Again, significant associations with clinical outcome were found after the acute phase. Increases in both interhemispheric functional connectivity (FC) and ipsilesional CST-FA were significantly correlated with the greater change of Fugl–Meyer Assessment (FMA) between weeks 1 and 4 post-stroke. In line with these findings, Mahmoud and colleagues [37] studied a cohort of 60 acute ischemic stroke patients within 2 days from onset. In patients with intact WM tracts, near-complete clinical recovery was noted. Moreover, the magnitude of FA reduction in the involved tracts was significantly associated with clinical score at admission and the 3-month clinical outcome. FA value in relation to motor recovery was also evaluated by Liu and colleagues [25] in their DTI study of 18 patients examined at weeks 1, 4, and 12 post-stroke. They reported positive correlations between FA values of contralesional medial frontal gyrus (MFG), thalamocortical connections, and FMA scores within 12 weeks following infarction. Besides CST, Takenobu and colleagues [28] studied the role of red nucleus in association with motor recovery in 10 patients who underwent DTI within 2 weeks and 1- and 3-months post-stroke. Significant positive correlation between the FA value of the red nucleus or dorsal pons cluster and Fugl–Meyer motor scale (FMMS) scores was observed, highlighting that microstructural alterations in the rubrospinal pathway may mediate motor recovery. With respect to upper extremity recovery, Doughty and colleagues [49] investigated the role of FA in the acute phase of stroke in relation to motor outcome at 3 months, using the Upper Extremity Fugl–Meyer (UE-FM) assessment. DTI was conducted in 58 patients within 80 h post-stroke, with ROIs located at the cerebral peduncle and a stretch of the CST caudal to each stroke lesion; the slope of the FA laterality and apparent diffusion coefficient (ADC) laterality indices of the nearest-5-slices (N5S) could predict 3-month UE-FM score weakly. In another study including 100 patients with ischemic stroke at the acute phase and applying DTI tractography to reconstruct both CST and commissural and associative WM tracts, Kulesh and colleagues found that the integrity (FA) of the integrity of the associative tracts of the affected hemisphere is more valuable than the microstructure of the intact hemisphere and the rFA for the prediction of global outcome whereas the integrity of the tracts of the intact hemisphere are important for the restoration of complex rehabilitation spheres, such as cognitive status and daily living and social skills [63].

Regarding aphasia recovery, Keser and colleagues [30] explored the integrity of arcuate fasciculus (AF) and frontal aslant tract (FAT) in 24 patients with left hemisphere ischemic stroke and consequent aphasia who underwent neuroimaging and language testing at acute and chronic time points. Acute right and left AF and FAT DTI values failed to correlate with recovery rate. On the contrary, longitudinal FA of the right AF was inversely correlated with naming recovery, providing evidence that reliance on left hemispheric components is linked with greater language recovery. With respect to perisylvian language networks, Forkel and colleagues [29] aimed to identify potential anatomic predictors of language recovery using DTI tractography. In their study, in the left hemisphere the only anatomical predictor of longitudinal aphasia was lesion size, whereas in the right hemisphere age and AF volume were associated with aphasia severity.

### 4.2. Prediction of Recovery Using Different DTI Parameters in Studies with Ischemic Cohorts: The Role of the FA Ratio

With regards to the FA ratio (rFA), Ali and colleagues [46] investigated 21 patients and demonstrated that an FA ratio between the affected and unaffected side under 0.8 at admission was associated with deficient motor recovery at discharge. Additionally, they visualized WM tracts using DTI tractography and found that patients with whole involvement of pyramidal tract exhibited higher NIHSS at discharge compared with the groups with intact and partial involvement. Similarly, Shaheen and colleagues [38] studied 45 middle cerebral artery (MCA) stroke patients and 17 controls and demonstrated that baseline FA and rFA were negatively associated with the NIHSS and Modified Rankin Score (mRS) and positively associated with Motricity Index (MI). In contrast to the aforementioned studies where DTI metrics at acute phase were predictive of outcome, Darwish and colleagues [54] examined 30 patients who underwent DTI on admission and 1 month post-stroke and found significant negative correlation between rFA at the rostral pons 1 month post-stroke and NIHSS score at 6 months. These findings also applied to FN in the CST ipsilateral to infarct. The prognostic value of rFA was also explored by Puig and colleagues [44] in a study of 89 MCA ischemic stroke patients who underwent DTI within 12 h, 3 days, and 30 days post-stroke onset. They illustrated that rFA at day 30 was the only independent predictor of long-term motor outcome. Similarly, Zhang and colleagues [60] studied 17 pontine infarct patients who underwent DTI at the acute phase and then consecutive examinations during a 6-month period and aimed to explore association with motor recovery. Positive correlation between the FMA scores on days 90 and 180 and rFA above the pons on day 14 were noted in line with these findings; Kwon and colleagues [56] sought to compare the predictability of early (1–14 days) and late (15–28 days) DTI by applying DTI tractography in relation to motor outcome at 6 months. Interestingly, CST integrity of the late scanning group could predict MI score, as opposed with the early group. Rong and colleagues [22] studied 3 medulla infarction patients with DTI and DTI tractography within 7, 14, and 30 days post-stroke onset and the FMA and Barthel index (BI) at each visit; two patients presented good motor recovery after 14 days and the FA values of their affected pyramidal tracts were slightly decreased, while they passed along periinfarct areas and their integrity was preserved in the medulla on DTI tractography. The most evident decrease in FA values along the affected CST was observed in the third patient, with right upper limb motor deficits presenting after 30 days. On DTI tractography, the affected pyramidal tract passed through the infarct and exhibited disruption in the medulla. Thus, it appears that the magnitude of impairment and sparing of periinfarct pyramidal tract compensation may constitute an important motor recovery mechanism.

### 4.3. Prediction of Recovery Using Different DTI Parameters in Studies with Ischemic Cohorts: The Role of MD

Regarding MD in stroke outcome prognosis, in the study of Liu and colleagues [32] 33 patients with acute subcortical stroke were investigated with DTI focused on the integrity of the inferior cerebellar peduncles (ICP) and lower limb FM assessment within 1 week, 4 weeks and 12 weeks post-stroke. Both MD and FA in contralesional ICP showed association with lower-limb FM score changes. Etherton and colleagues [61] studied a cohort of 42 patients to assess the role of DTI in early neurological improvement. Normal-appearing white matter (NAWM) MD was significantly lower in the group with early neurological improvement, and in multivariable logistic regression it was an independent predictor for early neurological improvement. 

### 4.4. Prediction of Recovery Using Different DTI Parameters in Studies with Ischemic Cohorts: The Role of AD

With regards to the AD, Moulton and colleagues [52] examined 28 thrombolysed patients and found that the strongest independent predictor of clinical outcome was the corona radiata AD ratio (rAD), correlating with motor NIHSS scores on day 7 and with mRS at 3 months. Interestingly, FA values could not be correlated with clinical recovery. In another study of 45 patients under thrombolysis, Moulton and colleagues [18] reported that rAD in CST predicted long-term motor recovery, whereas rAD in AF independently predicted a 3-month aphasia outcome, thus highlighting its potential as an efficient biomarker in the acute phase of stroke. Similarly, Groisser and colleagues [62] recruited 10 acute stroke patients with severe upper-limb involvement and examined the association of CST injury with motor recovery; acute decrease of CST AD has a prognostic utility in strength and fine motor functions in both the subacute and the chronic phase. Liu and colleagues [58] studied 22 patients and 22 controls and highlighted that changes in the FM scores were greater in those patients with higher changes in AD of the ipsilesional primary motor area. However, the only predictor of motor improvement within 12 weeks post-stroke was initial impairment or lesion volume.

### 4.5. Prediction of Recovery Using Different DTI Parameters in Studies with Ischemic Cohorts: The Role of Fiber Number Ratio

As for the rFN, Jang and colleagues [55] studied 31 pontine infarct patients using DTI at days 7–28 and clinical evaluation at 6 months using MI, a modified Brunnstrom classification (MBC), and functional ambulation category (FAC). The rFN and the CST area ratio significantly correlated with all 6-month motor outcomes. Surprisingly, rFA failed to reach significant association with all 6-month motor functions. Maraka and colleagues [57] studied 23 ischemic stroke patients with DTI, UE-FM and mNIHSS at the acute, subacute and chronic phase reported that rFN (affected/unaffected CST) showed strong correlation with the UE-FM score, whereas it was negatively correlated with mNIHSS at each phase of ischemic stroke. The prognostic utility of early rFNR was explored by Bigourdan and colleagues [24] in 117 patients assessed for motor recovery using the FMA score. The rFN was evaluated at 24 to 72 h and 1 year post-stroke. It was demonstrated that the rFN in acute phase correlated with the measurement conducted at 1 year and it was strongly predictive of motor outcome.

### 4.6. Prediction of Recovery Using Different DTI Parameters in Studies with Hemorrhagic Cohorts: The Role of FA

Regarding DTI in prediction of recovery in hemorrhagic stroke, the majority of studies focus on FA in CST. Ma and colleagues [35] applied DTI imaging in a cohort of 23 hemorrhagic stroke patients at different time points, including day 0. They evaluated outcome with motor function score (MFS) 90 days post-stroke. Significant association was noted between initial FA of the affected cerebral peduncle and MFS of day 90. Additionally, the initial FA value over 0.45 was a motor outcome predictor with high sensitivity and specificity. Similarly, Kuzu and colleagues [36] conducted a study on 23 patients with intracerebral hemorrhage (ICH) who underwent DTI five times for the study of cerebral peduncles, with the first scanning being within 3 days post-onset and outcome of motor function being assessed on day 90. In the good recovery group, mean FA on day 3 was significantly higher than in the poor recovery group and this value in the pathological side could predict motor recovery with sensitivity of 100% and a specificity of 77.8%. Similarly, Min and colleagues [48] studied 12 putaminal hemorrhage patients with DTI and clinical assessment within day 1 and 3 weeks, 3 months, and 6 months after the initial treatment and found that mean FA at the level of the pons in the affected side on day 1 and on 6 months was higher in the good outcome group than in the poor outcome group. They also reported significant association between the initial mean FA value and the sum of Brunnstrom motor recovery stage scores at 6 months. 

### 4.7. Prediction of Recovery Using Different DTI Parameters in Studies with Hemorrhagic Cohorts: The Role of FA Ratio

The rFA of cerebral peduncles (CP) was also investigated in the study of Wang and colleagues [64]. They demonstrated that within 3 days the ratio of rFA (affected/unaffected side) exhibited negative correlation with the paresis grading and mRS and positive correlation with the FIM scores at the end of follow-up. On the other hand, rFA at 2 weeks had positive correlation with the FIM and negative correlation with mRS scores and PG at the end of follow-up. Notably, as compared to the DTI within 3 days of ICH onset, the application of DTI at 2 weeks after ICH was superior to DTI within 3 days in terms of accurate prediction of motor outcome and daily living activities. In the study of Koyama and colleagues [43] including 32 patients with thalamic and putaminal hemorrhage, rFA values of the CP were significantly associated with Medical Research Council (MRC) scores, whereas the correlations with DTI values obtained for corona radiata/internal capsule were less significant. Similarly, another study Koyama and colleagues [41] reported a statistically significant association between rFA of CP and upper extremities function, which was stronger for the upper limb. Interestingly, rFA and FIM-motor scores were not correlated. On the other hand, in the study of Koyama and colleagues [42], rFA on cerebral peduncles were significantly associated with FIM-motor. The role of CP were also assessed by Tao and colleagues [50]; FA was measured within 4 days after onset at five slices below the level of the lesion on the affected and unaffected CST and in the CP along with rFA. Although rFA values at the CPs level were significantly lower in the poor functional outcome group, they were inferior to ICH score in predicting functional outcome. rFA was also explored in the prospective study of Cheng and colleagues [53] in 48 ICH patients who underwent DTI with a median time interval from onset of 7 days. Motor outcome was evaluated by MI at admission and after 1 and 3 months. It was found that lower rFA at the corona radiata predicted poor outcome at all time points of clinical evaluation. 

### 4.8. Prediction of Recovery Using Different DTI Parameters in Studies with Hemorrhagic Cohorts: The Role of Qualitative Assessment of CST Integrity

The role of CST integrity in relation to hand function was explored by Gong and colleagues [45] in a study of 75 hypertensive hemorrhage patients receiving DTI in approximately 3 weeks following stroke. It was shown that degree of CST integrity was negatively correlated with the Brunnstrom Recovery Staging-Hand (BRS-H) at 3 weeks and 3 months. In particular, patients with intact or complete disruption of CST failed to present substantial improvement in BRS-H at 3 months. On the contrary, those having partial CST impairment based on DTI were remarkably improved at 3 months in comparison to 3 weeks post-stroke.

### 4.9. Prediction of Recovery Using Different DTI Parameters in Studies with Both Ischemic and Hemorrhagic Cohorts: The Role of FA and the FA Ratio

In a study including both hemorrhagic and ischemic stroke patients, Imura and colleagues [47] evaluated the most efficient DTI parameters in predicting motor outcomes and activities of daily living function. They employed FA, FN, and ADC within 10 days post-stroke. Clinical outcome was re-assessed at 1 month. Only FA of the affected CST exhibited significant correlation with the motor outcome and activities of daily living function within 10 days and at 1 month post-onset. In a similar cohort, Nakashima and colleagues [39] examined 17 patients with DTI and voxel-based morphometry and assessed clinical outcome at 3 months using FMA and the Motor Activity Log (MAL). In patients with incomplete CST disruption in tractography, rFA of the bilateral CP showed significant correlation with FMA, amount of use, and quality of movement. The rFA on CP was also examined in the study of Koyama and colleagues [40] who included 80 patients with hemorrhagic and ischemic stroke and DTI on days 14–21. Both the hemorrhagic and the infarct groups showed similar patterns of statistically significant correlations between rFA and outcome measures. Interestingly, such similarity did not apply to FIM-motor. In agreement with these findings, Okamoto and colleagues [34] investigated a cohort with both ischemic and hemorrhagic stroke patients and concluded that higher rFA values of the PLIC at admission to the recovery rehabilitation unit were associated with better outcomes of upper extremity function.

### 4.10. Methodological Considerations

The review of the published DTI studies reveals diverse methodological approaches. The majority of DTI studies, relying on 1.5 T or 3.0 T field strength, adopt a multi-ROI protocol for the reconstruction of WM tracts and only few capitalize on recent advances in whole-brain DTI. The vast majority of studies apply less than 30 gradient directions with only few studies using equal or more than 30 gradient directions. The anatomical accuracy of DTI tractography is inherently constrained since inferring information for fiber direction based on the water diffusion profile is a complex, undetermined inverse problem [65]. There are several pitfalls and sources of errors that may limit the accuracy of the tractography output. Of note, these limitations can emerge at any stage, including image acquisition, algorithms applied for tractography (deterministic vs. probabilistic, different deterministic algorithms), local voxel-wise reconstruction and tracking streamlines. Diffusion MRI is prone to artifacts that are related to susceptibility gradients affecting echo planar imaging acquisition, head motion, and eddy currents. All of these can affect the orientation estimates, diffusion indices, and geometric structure of the pathways which can result in anatomically incorrect tractography [66,67,68,69,70]. In addition, data acquisition features (e.g., magnetic field, signal-to-noise ratio, number of diffusion-encoding directions, b-values, voxel resolution) also affect fiber reconstruction [71,72,73]. Furthermore, each voxel can contain a large number of axons with many potential complex geometric configurations. Fibers with crossing, kissing, fanning and/or curving configurations are difficult to reconstruct specifically using early single-tensor deterministic algorithms (i.e., Fiber Assignment by Continuous Tracking), thus resulting in incorrect estimates of fiber orientation and false-positive or false-negative reconstructed fibers [74]. Of note, different algorithmic fiber tracking methodologies as well as the particularities of different WM fiber bundles affect the final reconstructed fibers, with some algorithms used for DTI tractography being less sensitive to the crossing and kissing fibers problem (e.g., reconstruction of CST, corpus callosum forceps major and forceps minor, and superior longitudinal fasciculus) [75]. Additionally, the tracking process is also subject to biases and/or inaccuracies due to the application of manual vs. automated tractography methods (with the former also being subject to raters’ knowledge of brain anatomy and experience regarding ROI selection and interpretation of false-positive and/or false-negative fibers) or choice of tracking parameters (e.g., seeding and stopping criteria for FA thresholds, curvature thresholds, step size, and fiber length) [76,77,78]. These methodological points have been well-documented [79] and their thorough consideration in stroke patients [80,81] explain different interpretations of DTI biomarkers both in acute and chronic stroke. The most evaluated WM tract is the pyramidal tract (either a single tract or specific regions located across the tract). Strikingly few studies have evaluated non-motor tracts, with most of them examining language-related tracts. Other WM tracts that might be affected in stroke and demonstrate a significant role in other cognitive functions (e.g., memory, perception) are not examined and clinical outcome with regards to these functions are not reported.

### 4.11. Common Shortcomings

The review of the published DTI studies also reveals common study limitations. Most of the published DTI studies in stroke patients only report single-modality changes; functional changes (i.e., fMRI or TMS) are not or only seldom evaluated. Some recent studies describe additional functional alterations but direct associations between structural and functional neuroimaging metrics are not consistently reported or not performed. Most hemorrhagic studies are cross-sectional, which preclude the assessment of longitudinal changes, ceiling, and flooring effects. The inclusion of multiple timepoints, although often difficult to apply in clinical practice, might also reveal the existence of linear and/or non-linear changes. Cross-sectional studies merely offer a snapshot of structural changes in WM tracts and patients are often in a different reorganization/regeneration stage of their post-stroke trajectory, which can be further complicated by pharmacological and non-pharmacological interventions. The latter are not comprehensively described in longitudinal studies and need to be addressed in future prospective studies. The admixing of patients with ischemic and hemorrhagic stroke should also be carefully considered when interpreting different findings. A requisite of future DTI studies is a prospective, multi-timepoint longitudinal design with a large and relatively homogenous clinical profile at enrolment and uniform follow-up intervals. 

### 4.12. Future Directions

DTI and tractography need to be integrated into routine imaging protocols in stroke patients to establish their detection sensitivity, relative advantages, and monitoring and predictive potential. Future longitudinal studies should highlight the role of DTI as a viable clinical tool for monitoring and predicting patients’ functional outcomes not only with regards to motor and language functions but also other cognitive functions (e.g., memory), and detecting response to therapy, including different rehabilitation techniques [e.g., repetitive TMS (rTMS); transcranial direct current stimulation (tDCS)]. The practical demands of clinical imaging require relatively short acquisition times, transparent and reliable data interpretation processes, and ease of harmonizing protocols across clinical sites to conduct multicenter studies. There is a relative urgency to shift the emphasis from descriptive, qualitative studies to the development of protocols with practical clinical utility that can be applied in large sample sizes and provide accurate biomarkers for the prediction of stroke recovery. Finally, its potential as a putative monitoring biomarker for pharmacological and non-pharmacological interventions in stroke patients should be carefully evaluated to further identify both reorganization and degeneration processes.

## 5. Conclusions

In conclusion, the results of the present review are indicative of the utility of DTI and specific parameters (e.g., FA, FA ratio, diffusivity values, and fiber number ratio) for tracking longitudinal changes and identifying prognostic correlates in acute and hyperacute stroke patients. However, it is worth mentioning that additional efforts are needed to translate the insights gained from DTI studies in stroke patients into practical applications in clinical settings and routine clinical practice. Of note, ROC curves and various machine-learning frameworks in structural imaging in other neurological and psychiatric groups suggest that DTI may have a role in predicting patients’ outcomes with adequate accuracy at a single-patient level, thus providing valuable information for patients’ therapeutic management and both short- and long-term outcomes. 

## Data Availability

All data discussed within this manuscript are available on PubMed.

## Abbreviations

Action Research Arm Test (ARAT); activities of daily living (ADL); AD ratio (rAD); apparent diffusion coefficient (ADC); arcuate fasciculus (AF); axial diffusivity (AD); Barthel index (BI); Berg Balance Scale (BBS); Brunnstrom Recovery Stage (BR); Brunnstrom Recovery Staging-Hand (BRS-H); cerebral peduncles (CP); corticospinal tract (CST); diffusion tensor imaging (DTI); diffusion weighted imaging (DWI); magnetic resonance imaging (MRI); FA ratio (rFA); fiber number (FN); fiber number ration (rFN); fractional anisotropy (FA); frontal aslant tract (FAT); Fugl-Meyer Assessment (FMA); Fugl-Meyer motor scale (FMMS); functional ambulation category (FAC); functional connectivity (FC); Functional Independence Measure (FIM); Hauser Ambulation Index (HAI); inferior cerebellar peduncles (ICP); intracerebral hemorrhage (ICH); mean diffusivity (MD); medial frontal gyrus (MFG); Medical Research Council (MRC); middle cerebral artery (MCA); Modified Barthel Index (MBI); modified Brunnstrom classification (MBC); motor evoked potentials (MEP); motor items of National Institutes of Health Stroke Scale (mNIHSS); Modified Rankin Score (mRS); Motor Activity Log (MAL); motor component of the FIM (FIM-motor); motor function score (MFS); Motricity Index (MI); Montreal Cognitive Assessment Scale (MoCA); National Institutes of Health Stroke Scale (NIHSS); nearest 5 slices (N5S); normal appearing white matter (NAWM); posterior limbs of the internal capsule (PLIC); Preferred Reporting Items for Systematic Reviews (PRISMA); radial diffusivity (RD); Rivermead Mobility Index (RMI); region of interest (ROI); repetitive TMS (rTMS); resting-state functional MRI (rs-fMRI); transcranial direct current stimulation (tDCS); transcranial magnetic stimulation (TMS); Upper Extremity Fugl-Meyer (UE-FM); white matter (WM); length of hospital stay (LOS); Jebsen Hand Function Test (JHFT); centrum semiovale (CSO); genum of the corpus callosum (CCg); splenium of the corpus callosum (CCs); precentral gyrus (PrCG); corpus callosum (CC); cingulum bundle (CB); laterality index (LI); tract-based spatial statistics (TBSS); superior longitudinal fasciculus (SLF); inferior fronto-occipital fasciculus (IFOF); inferior longitudinal fasciculus (ILF); uncinate fasciculus (UF); Jebson-Taylor Test (JTT); Aphasia Handicap Score (AHS); Boston Naming Test (BNT); functional connectivity (FC); anterior thalamic radiation (ATR); cingulate gyrus (CCG); cingulate hippocampus (CH); forces major (FMAJ); forces minor (FMIN); superior longitudinal fasciculus (temporal part) (SLF-TP); Nine Hole Peg Test (NHPT).
